# Betulinic acid augments the inhibitory effects of vincristine on growth and lung metastasis of B16F10 melanoma cells in mice

**DOI:** 10.1038/sj.bjc.6601746

**Published:** 2004-03-23

**Authors:** N Sawada, K Kataoka, K Kondo, H Arimochi, H Fujino, Y Takahashi, T Miyoshi, T Kuwahara, Y Monden, Y Ohnishi

**Affiliations:** 1 Department of Oncological and Regenerative Surgery, School of Medicine, The University of Tokushima, Tokushima 770-8503, Japan; 2 Department of Molecular Bacteriology, Graduate School of Medicine, The University of Tokushima, Tokushima 770-8503, Japan

**Keywords:** synergistic antitumour effect, betulinic acid, vincristine, cell cycle arrest, G1 phase

## Abstract

We examined the antitumour effect of a combination of betulinic acid (BA) and vincristine (VCR) on murine melanoma B16F10 cells *in vitro* and *in vivo*. Betulinic acid, a pentacyclic triterpene, showed a synergistic cytotoxic effect on melanoma cells by combinational use of VCR. Betulinic acid and VCR induced cell cycle arrest at different points (BA at G1 phase and VCR at G2/M phase) and caused apoptosis in B16F10 melanoma cells. In the *in vivo* study, VCR inhibited metastasis of tumour cells to the lung. The addition of BA to VCR augmented suppression of the experimental lung metastasis of melanoma cells in C57BL/6 mice. The number of lung nodules of more than 1 mm in diameter in mice treated with BA and VCR was less than that in mice treated with VCR alone. These results suggest that BA is an effective supplement for enhancing the chemotherapeutic effect on malignant melanoma.

When multiple metastases are found at the time of clinical diagnosis, surgical operations are usually not performed, and the patients are treated mainly by radiotherapy or chemotherapy with various anticancer drugs. To investigate the effects of anticancer drugs on tumour cells and their lung metastasis, the experimental lung metastasis model by Fidler and Kripke (1977) has been used (Miyoshi *et al*, 2000). Although anticancer drugs are widely used for cancer therapy, a sufficient quantity of the drug for treatment cannot be administered in many cases due to severe side effects.

Malignant melanoma is difficult to cure. Since melanoma cells have the ability to spread to distal organs, various anticancer drugs have been used in combination to try to improve the therapeutic effect on malignant melanoma. For example, lovastatin has been shown to potentiate the antitumour activity of doxorubicin in murine melanoma (Feleszko *et al*, 2002), and exisulind in combination with docetaxel has been shown to inhibit the growth and metastasis of human lung cancer (Chan *et al*, 2002). Recently, the antitumour effect of combinational therapy using an anticancer drug and a phytochemical has been studied (Darzynkiewicz, 1995; Christensen and LeBlanc, 1996; Singh *et al*, 2002). Phytochemicals such as flavonoid have relatively low levels of toxicity (Singh *et al*, 2002) and can induce apoptosis or cell cycle arrest in tumour-derived cells (Darzynkiewicz, 1995). We previously reported that an extract of a medicinal plant lemon grass containing various phytochemicals inhibited the formation of azoxymethane-induced DNA adducts and aberrant crypt foci in the rat colon (Suaeyun *et al*, 1997).

Betulinic acid (BA), a pentacyclic triterpene isolated from the root bark of *Morus australis* or *Clerodendrum mandarinorum* and many other medicinal plants (Zhu *et al*, 1996; Ko *et al*, 1997), has recently been reported to have cytotoxic activity against several tumour cell lines (Pisha *et al*, 1995; Fulda *et al*, 1997, 1998; Wick *et al*, 1999; Kwon *et al*, 2002; Wachsberger *et al*, 2002). Betulinic acid is a novel experimental antineoplastic agent for human melanoma cells *in vitro* (Pisha *et al*, 1995; Wachsberger *et al*, 2002). Betulinic acid also induces apoptosis in neuroectodermal tumour and glioma cells (Fulda *et al*, 1997, 1998; Wick *et al*, 1999). Furthermore, BA has been shown to inhibit growth factor-induced angiogenesis *in vitro* (Kwon *et al*, 2002).

Vincristine (VCR) is one of the major chemotherapeutic agents used for the treatment for malignant melanoma. Vincristine showed antitumour effects on B16 melanoma cells, but a high dose of VCR induces severe bone marrow toxicity (Morrow *et al*, 1987). In this study, we evaluated the effectiveness of combination therapy with VCR and BA for malignant melanoma by examining the synergistic cytotoxic activity against B16F10 melanoma cells *in vitro* and *in vivo*.

## MATERIALS AND METHODS

### Chemicals

Betulinic acid, VCR, annexin V–FITC and propidium iodide (PI) solution was obtained from Wako Pure Chemical Industries, Ltd, Osaka, Japan. Triton X-100, proteinase K, agarose and boric acid were obtained from Sigma Chemical Co., St Louis, MO, USA. Betulinic acid and VCR were dissolved in dimethyl sulphoxide (DMSO) and saline, respectively. They were diluted with saline to appropriate concentrations and stored in a refrigerator at 4°C until use.

### Animal model

Female C57BL/6 mice (9–10 weeks old) obtained from SLC Japan (Hamamatsu, Japan) were housed in plastic cages with sawdust bedding and given food and water *ad libitum*. The room in which the mice were kept, in the Institute of Animal Experimentation, School of Medicine, The University of Tokushima, Japan, was environmentally controlled at a temperature of 23±2°C and humidity of 55±10% and with a 13-h light/11-h dark cycle. All animal experiments were carried out with approval by the Institutional Animal Care and Use Committee of the University of Tokushima School of Medicine, and met the standards required by the United Kingdom Coordinating Committee on Cancer Research (UKCCCR) guidelines (Workman *et al*, 1998).

### Cell culture conditions

B16F10 melanoma cells were purchased from the American Type Culture Collection (Virginia, USA) and were maintained in DMEM medium (Nissui Pharmaceutical Co., Japan) supplemented with 5% calf serum (Gibco Co., NY, USA), 5% horse serum (Gibco), penicillin–streptomycin mix (Gibco) and gentamycin (Gibco). B16F10 melanoma cells were passaged every 3–4 days.

### Annexin V-based apoptosis analysis

B16F10 melanoma cells (1 × 10^5^) were cultured in 25 cm^2^ tissue culture plates for 24 h before the treatment with test reagents. Betulinic acid at concentrations of 1, 2.5 and 5 *μ*M or VCR at a concentration of 1 nM was added, and the cells were incubated for a further 24 h. After the incubation, the cells were trypsinised, washed with phosphate-buffered saline (PBS), and suspended in 1 ml of a calcium-containing binding buffer. Then 5 *μ*l of annexin V–FITC and 5 *μ*l of PI were added to the cell suspensions. After 10-min incubation at room temperature, early apoptotic cells (annexin V positive and PI negative) and late apoptotic/necrotic cells (annexin V positive and PI positive) as well as living cells (annexin V negative and PI negative) were detected by a flow cytometer (Coulter Epics XL-MCL, Beckman Coulter, Tokyo, Japan). The data were analysed using CellQuest software. The early and late apoptotic cells (annexin V positive) were defined as the apoptotic population.

### DNA fragmentation-based apoptosis analysis

Total DNA was extracted according to the method of Lee and Shacter (1999). Briefly, B16F10 melanoma cells (2 × 10^6^) were cultured in 60 cm^2^ tissue culture plates for 24 h before the treatment with test reagents. Betulinic acid at concentrations of 1, 2.5 and 5 *μ*M was added, and the cells were incubated for a further 24 h. Cells were harvested, washed with PBS and lysed in buffer containing 10 mM Tris-HCl (pH 7.8), 10 mM EDTA, 0.5% Triton X-100, 200 *μ*g ml^−1^ RNaseA and 200 *μ*g ml^−1^ proteinase K. After DNA was precipitated with isopropanol, it was then resuspended in a Tris-EDTA solution. Samples were resolved by electrophoresis on 2% agarose gels and visualised by ethidium bromide staining and UV transillumination.

### Cell cycle distribution analysis

The effect of BA or VCR on cell cycle distribution *in vitro* was determined by using a flow cytometer. B16F10 melanoma cells (1 × 10^6^) were cultured in 25 cm^2^ tissue culture plates for 24 h before the treatment with test reagents. After 24 or 36 h of incubation with BA at concentrations of 1, 2.5 and 5 *μ*M or VCR at a concentration of 1 nM, B16F10 melanoma cells were fixed with 99% ethanol, treated with RNaseA and stained with PI solution containing RNase A. A flow cytometer was used to determine the percentages of cells in G0/G1, S and G2/M phases of the cell cycle.

### MTS assay

The standard MTS (3-(4,5-dimethylthiazol-2-yl)-5-(3-carboxymethoxyphenyl)-2-(4-sulphophenyl)-2H-tetrazolium, innersalt solution) (Takara Co., Kusatsu, Japan) assay was performed to examine cytostatic/cytotoxic effects of BA and/or VCR on B16F10 melanoma cells according to the manufacturer's instructions. Briefly, tumour cells (5 × 10^3^ cells) were seeded in 96-well microtiter plates (Becton Dickinson Co., Franklin Lakes, NJ, USA). After an overnight incubation at 37°C in 5% CO_2_, serially diluted solutions of BA, VCR or both were added in triplicate to a final volume of 200 *μ*l. After incubation for 24 h, the medium was removed, and the cells were washed twice with culture medium and resuspended in 200 *μ*l of culture medium. Then, 20 *μ*l of MTS was added to each well, and the plates were incubated at 37°C for 1 h. The absorbance of each well was read at 450 nm on a microplate reader (microplate reader: MTP-120, Corona Co., Katsuta, Japan). The relative viability was calculated as follows: relative viability=[(experimental absorbance−background absorbance)÷(absorbance of untreated controls−background absorbance)] × 100%.

### Drug interaction analysis

Synergistic interaction between BA and VCR was determined by using isobologram analysis as described in detail previously (Berenbaum, 1981; Sora *et al*, 1994). Briefly, inhibition of cell proliferation was determined as described above. Equieffective concentrations were used for the analysis. The interaction index for the two-drug combination was computed by the following equation: interaction index=BA*c*/BA*e*+VCR*c*/VCR*e*, where BA*e* and VCR*e* are concentrations of BA and VCR, respectively, that inhibit cell proliferation to 40% of the control level when used alone, and BA*c* and VCR*c* are concentrations of BA and VCR, respectively, that produce the same effect when used in combination. According to this method, an interaction index of less than 1.0 indicates synergistic interaction between two drugs. An interaction index of more than 1.0 indicates antagonism, and an index of 1.0 indicates additive interaction.

### Effects of BA and VCR and their combination on tumour development in C57BL/6 mice injected with B16F10 melanoma cells

**Figure 1:**
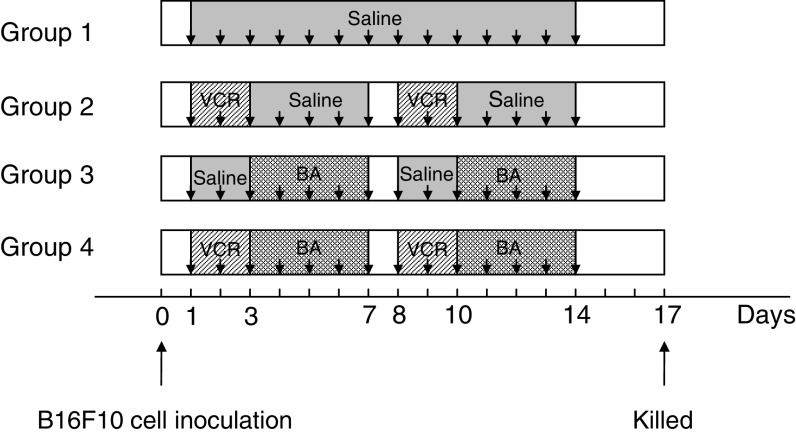
Experimental protocol for animal experiment. Arrows indicate the series of injection of saline, BA (BA, i.p., 10 mg kg^−1^) or VCR (VCR, i.p., 0.065 mg kg^−1^). Mice in groups 1, 2 and 3 were administered saline, VCR and BA, respectively. Mice in group 4 were administered both VCR and BA.

On day 0, B16F10 melanoma cells (1 × 10^5^ cells/0.1 ml medium) were injected into the tail veins of anaesthetised 9- to 10-week-old female C57BL/6 mice. Tumour-bearing mice were divided into four groups (five mice in each group) and treated intraperitoneally (i.p.) with saline (0.1 ml), BA (10 mg kg^−1^ day^−1^), VCR (0.065 mg kg^−1^ day^−1^) or a combination of both drugs (BA, 10 mg kg^−1^ day^−1^; VCR, 0.065 mg kg^−1^ day^−1^) (Figure 1

). The dose of BA for the treatment of mice was decided by referring to a previous report (Pisha *et al*, 1995). Body weights of mice were recorded every 4 days to determine whether the treatment influenced health status. The mice were killed by cervical dislocation under anaesthesia with diethyl ether 17 days after inoculation of tumour cells. The lungs were removed, and two independent observers determined the numbers and sizes of lung metastatic nodules.

### Data analysis

All data were statistically analysed by analysis of variance. *P*<0.05 was considered to be statistically significant.

## RESULTS

### Effects of VCR and/or BA on the growth of B16F10 melanoma cells *in vitro*

**Figure 2:**
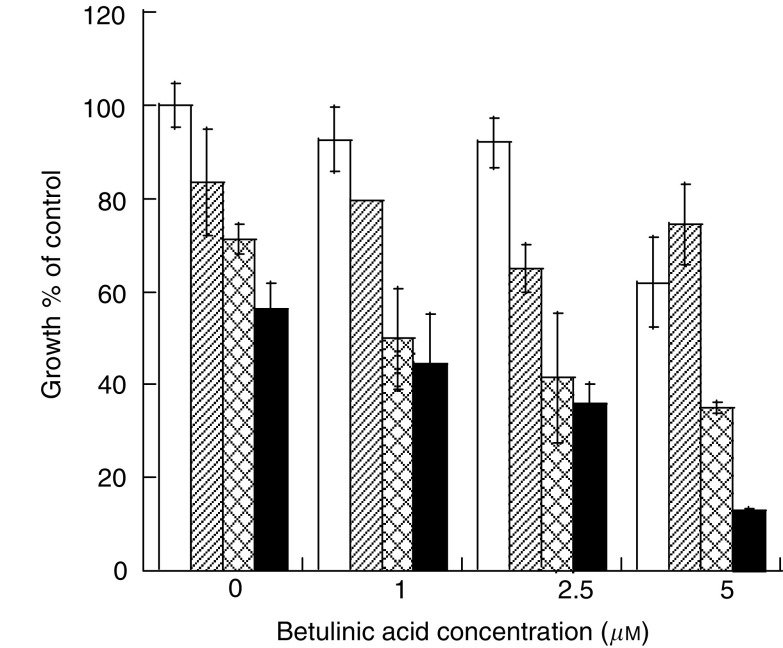
Effects of VCR and/or BA on the growth of B16F10 cells. Concentrations of VCR were 0 nM (open box), 0.1 nM (hatched box), 1.0 nM (latticed box) and 10 nM (closed box). Cytostatic/cytotoxic effects were tested by MTS assays and expressed as the percentage of the control growth (% of untreated control). Bars, mean±s.d.

Vincristine is one of the major chemotherapeutic agents for patients with malignant melanoma. To determine whether treatment with a combination of VCR and BA has a greater inhibitory effect than does treatment with VCR or BA alone, we examined the inhibitory effects of VCR alone, BA alone and the combination of them using MTS assays. When the growth level of untreated cells was regarded as 100%, the growth levels of cells treated with 0.1, 1 and 10 nM VCR were 83.4, 71.2 and 56.2%, respectively (Figure 2

). Vincristine inhibited the growth of B16F10 cells in a dose-dependent manner. In addition, BA also inhibited the growth of B16F10 cells in a dose-dependent manner (1 *μ*M, 92.5%; 2.5 *μ*M, 91.9%; 5 *μ*M, 62.0%). Combinational treatment with 1 or 10 nM VCR and BA resulted in the inhibition of the growth of B16F10 cells in a dose-dependent manner. The combination of 10 nM VCR and 5 *μ*M BA greatly inhibited the growth of cells (12.9%).

### Induction of apoptosis in B16F10 cells by VCR and BA

**Figure 3:**
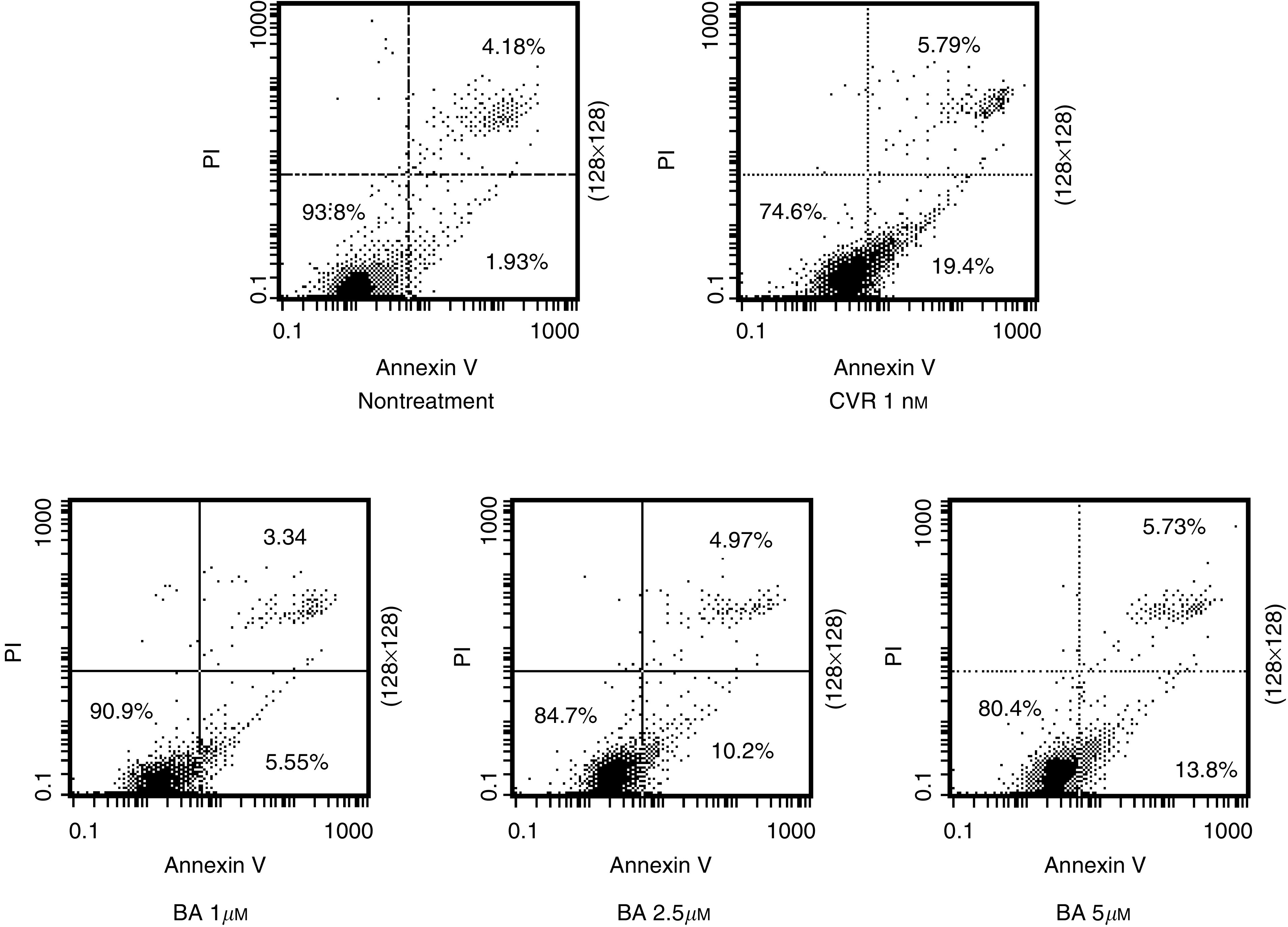
Apoptosis assay of B16F10 cells treated with VCR or BA. Flow cytometric analysis of apoptosis in B16F10 cells was performed by treatment with VCR (1 nM) and BA (1, 2.5 or 5 *μ*M) for 24 h and by staining with annexin V–FITC and PI.

**Figure 4:**
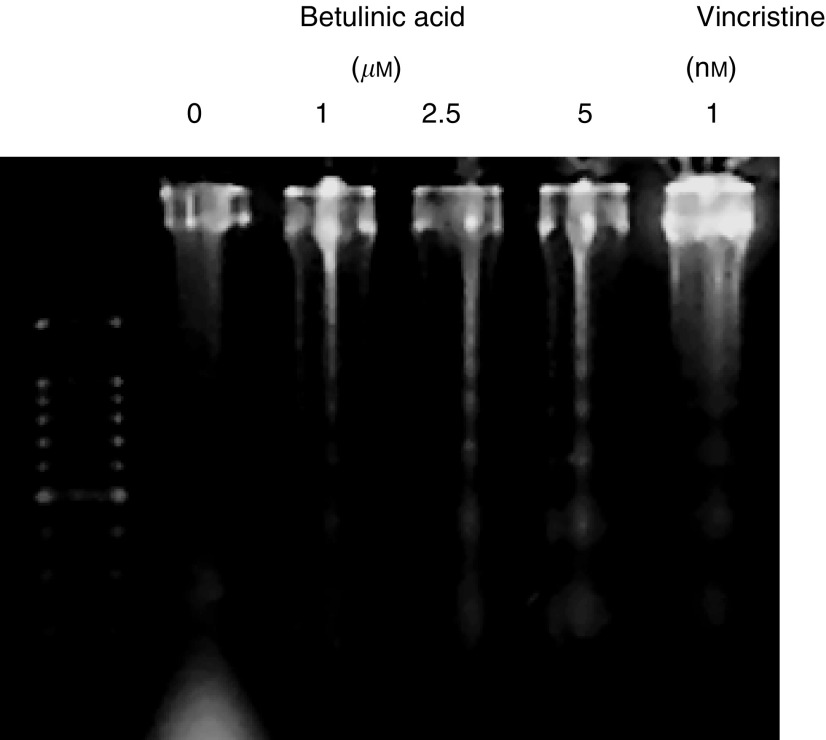
DNA fragmentation analysis of apoptosis in B16F10 cells treated with BA.

B16F10 melanoma cells were incubated in DMEM medium with VCR at 1 nM or BA at three different concentrations (1, 2.5 and 5 *μ*M) for 24 h. The percentage of apoptotic cells was determined as described in Materials and methods. The apoptotic population of untreated cells was 6.1%, while that of cells treated with 1 nM VCR was 25.2% (Figure 3

). The apoptotic populations of cells treated with 1, 2.5 and 5 *μ*M BA alone were 8.9, 15.2 and 19.5%, respectively. Moreover, the induction of apoptosis was evaluated by DNA fragmentation (Wyllie, 1980). DNA ladder formation was detected (Figure 4)

, and the fragments were stained in a dose-dependent manner. These findings indicate that BA induced apoptosis in a dose-dependent manner.

### Determination of the synergistic effect of VCR and BA using isobologram analysis

**Figure 5:**
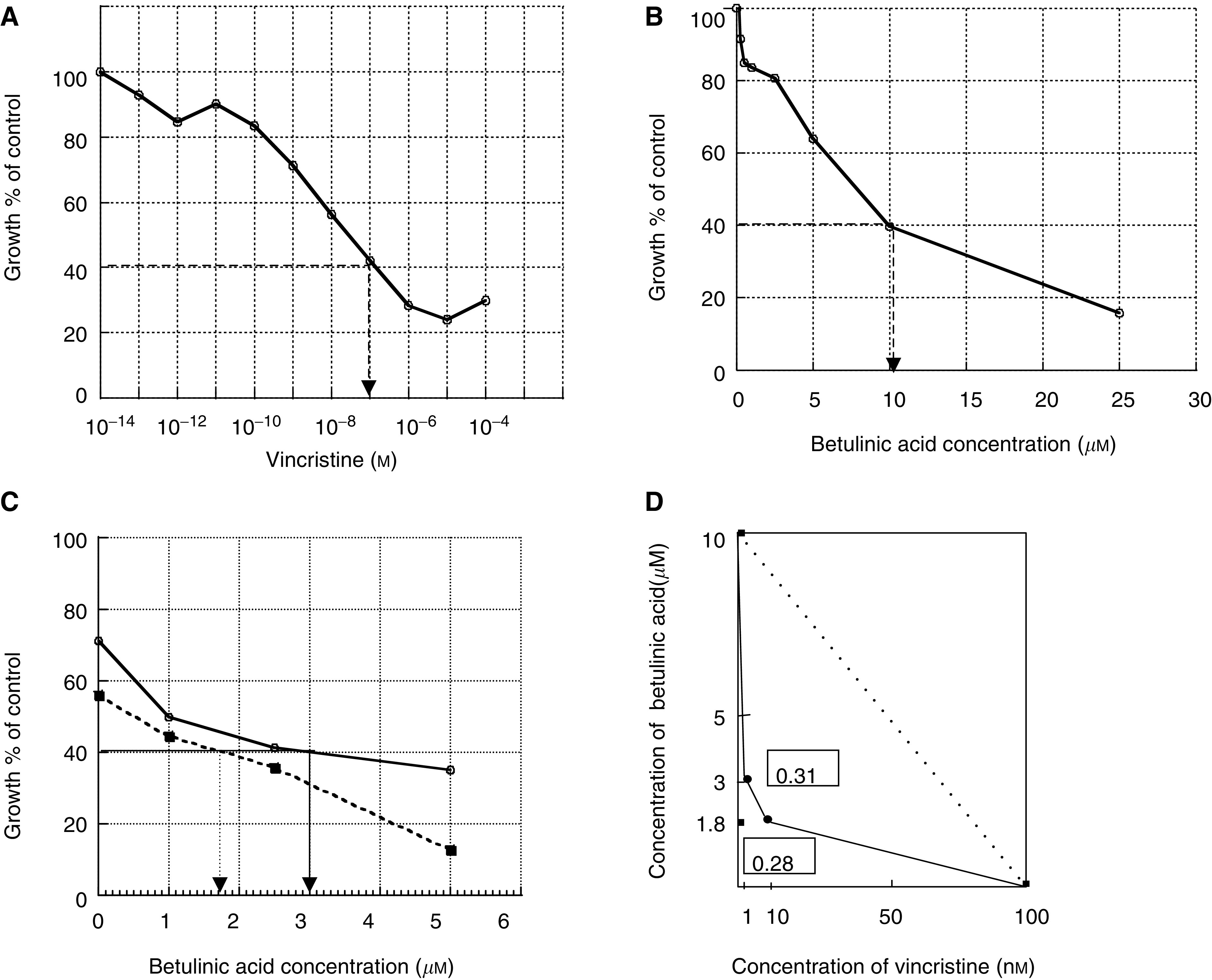
Effects of VCR and/or BA on the growth of B16F10 cells. Cytostatic/cytotoxic effects were tested by MTS assays and expressed as the percentage of the control growth (**A**–**C**). Solid and dotted lines show 1 and 10 nM VCR, respectively (**C**). The isobologram depicts the interaction between BA and VCR in inhibiting the growth of B16F10 cells (**D**). The solid line shows concentrations of both drugs required to inhibit cell growth to 40% (IC_40_). The dotted line shows concentrations of drugs required to produce the same degree of growth inhibition as that in the case of interactions being additive. The numbers in boxes are interaction indices.

To clarify the synergistic effect of VCR and BA on inhibition of the growth of B16F10 cells, we used isobologram analysis by the method of Berenbaum (1981). Treatment with VCR or BA resulted in inhibition of the growth of B16F10 cells in a dose-dependent manner (Figure 5a and b

). The inhibitory concentration (IC_40_) of VCR alone and that of BA alone showing 40% of the control growth level were 100 nM and 10 *μ*M, respectively (Figure 5a and b). When IC_40_ was determined by combinational use of both drugs, a combination of 10 nM VCR and 1.8 *μ*M BA, and a combination of 1 nM VCR and 3 *μ*M BA showed 40% growth of the control (Figure 5c). The interaction indices calculated by the equation shown in Materials and methods were 0.28 and 0.31, which are less than 1.0 (Figure 5d). The results of isobologram analysis showed that treatment with a combination of VCR and BA synergistically inhibited the growth of B16F10 cells.

### Induction of G2/M arrest and G1 arrest by VCR and BA, respectively

Cell cycle distribution analysis was performed to clarify the mechanism of synergic inhibitory action on B16F10 melanoma cells. B16F10 cells were incubated with VCR and BA for 24 or 36 h in the same manner as in the cell apoptosis assay. The proportions of cells in the G2/M phase that had been treated with 1 nM VCR for 24 and 36 h were 29.3 and 79.8%, respectively (untreated group: 14.5% after 24 h and 10.0% after 36 h) (Table 1

**Table 1:** Cell cycle distribution of B16F10 cells treated with VCR or BA

	**Cell cycle distribution (%)**					
	**G0/G1^a^**		**S^a^**		**G2/M^a^**	
**Treatment**	**24 h^b^**	**36 h^b^**	**24 h^b^**	**36 h^b^**	**24 h^b^**	**36 h^b^**
None	74.1	80.7	11.4	9.3	14.5	10.0
VCR (1 nM)	62.7	20.2	8.0	0.0	29.3	79.8
BA (1 *μ*M)	78.8	93.3	2.0	5.6	19.2	1.1
BA (2.5 *μ*M)	86.1	93.1	3.0	2.1	10.9	4.8
BA (5 *μ*M)	95.5	93.4	0.0	2.2	4.4	4.4

VCR=vincristine; BA=betulinic acid.

a Cell cycle phase.

b Incubation time.

), indicating that VCR induces cell cycle arrest at the G2/M phase. The population of G0/G1 cells treated with BA for 24 h increased in a dose-dependent manner (1 *μ*M, 78.8%; 2.5 *μ*M, 86.1%; 5 *μ*M, 95.5%). Moreover, the cell population in the G0/G1 phase after 36 h of treatment was constant (93%) regardless of the concentration (untreated group: 80%), indicating that BA induces cell cycle arrest at the G1 phase. These results demonstrated that VCR and BA induce cell cycle arrest at different stages.

### Antimetastatic effects of VCR, BA and their combination in C57BL/6 mice treated with B16F10 melanoma cells

**Figure 6:**
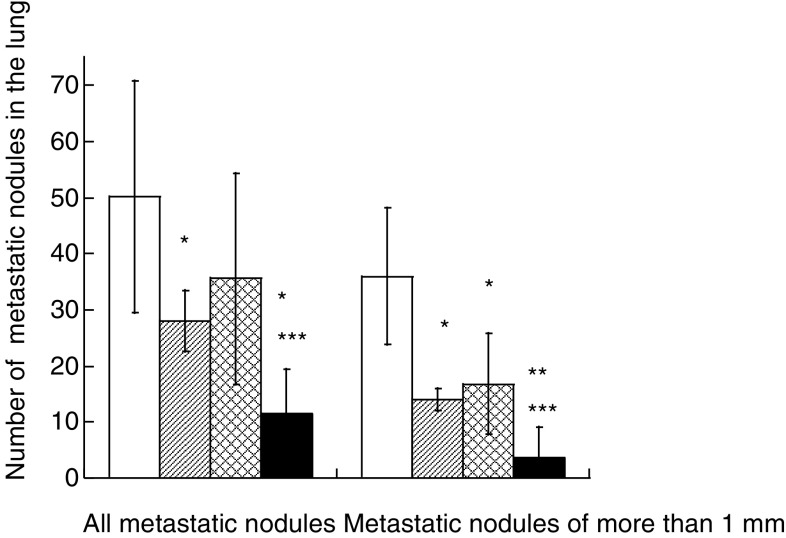
Effects of VCR and/or BA on experimental metastasis in mice. Mice were treated with a solvent (open box), 0.065 mg kg^−1^ VCR (hatched box), 10 mg kg^−1^ BA (latticed box), and both drugs (closed box) as described in Materials and methods. The numbers of tumour nodules were evaluated with classification by size (all sizes and more than 1 mm in diameter). ^*,**^Significantly different from the control group (*P*<0.05 and *P*<0.0001). ^***^Significantly different from the VCR group and the BA group (*P*<0.05).

To determine whether the augmenting effect of BA on the cytotoxicity of VCR against B16F10 melanoma cells can be reproduced *in vivo*, animal experiments were performed according to the schedule shown in Figure 1. B16F10 cells formed 50.2±20.7 nodules in the lung in the nontreatment group, whereas they formed 28.0±5.4, 35.6±18.8 and 12.4±9.5 nodules in the VCR, BA and combinational administration groups, respectively (Figure 6

). Treatment with VCR (0.065 mg kg^−1^ day^−1^) alone or in combination with BA (10 mg kg^−1^ day^−1^) significantly suppressed the experimental metastasis. B16F10 melanoma cells formed 36.0±12.2 large nodules of more than 1 mm in diameter in the lung in the nontreatment group, whereas they formed 14.0±2.0, 16.8±9.0 and 3.6±5.5 large nodules in the VCR, BA and combinational administration groups, respectively. The ratios of the number of large nodules to total metastatic nodules in the lung in the nontreatment group and the VCR, BA and combinational administration groups were 71.7, 50.0, 47.2 and 29.0%, respectively, indicating that combinational treatment with VCR and BA significantly suppresses the growth of B16F10 melanoma cells after their colonisation in the lung. No weight loss was observed in any mice treated with VCR, BA or both reagents (data not shown).

## DISCUSSION

In most cases of malignant melanoma, distant metastasis is found at the time of clinical diagnosis. It has been reported that the median survival time of stage IV melanoma patients is about 6–7.5 months, the 5-year survival rate being approximately 6% (Bajetta *et al*, 2002). Lung metastasis of melanoma cells from primary lesions is an especially important factor influencing the prognosis of patients. Therefore, the main treatment for malignant melanoma is various chemotherapeutic agents. Various anticancer drugs, including bleomycin, VCR, lomustine and dacarbazine, have been used in recent years for treating patients with melanoma (Wöll *et al*, 1999; Kivela *et al*, 2003). However, severe side effects of the chemotherapeutic agents have limited the application of high-dose chemotherapy or combination therapy with different anticancer drugs for many cases of malignant melanoma (Bajetta *et al*, 2002).

Combinational treatment with different drugs that induce different cell cycle arrest results in apoptosis in tumour cells (Darzynkiewicz, 1986; Bhuyan and Groppi, 1989; Ko *et al*, 1997). It was thought that treatment with the combination of BA and VCR had a synergistic cytotoxic effect on tumour cells because treatment with BA alone and treatment with VCR alone induced cell cycle arrest in different phases. Although BA did not induce cell cycle arrest in glioma cells (Wick *et al*, 1999), we found that BA induced the accumulation of B16F10 melanoma cells in the G1 phase after 24 h of coincubation in a dose-dependent manner. Moreover, BA induced the accumulation of 93% of B16F10 cells in the G1 phase after 36 h of coincubation. It is expected that elucidation of the mechanism by which BA inhibits the growth of B16F10 melanoma cells will lead to the establishment of effective combinational regimens with other antitumour drugs with different cytotoxic mechanisms for the treatment of malignant melanoma. It has been reported that BA enhanced the levels of BAX and BCL-2 proteins in glioma cells and induced apoptosis in those cells (Wick *et al*, 1999). BA induces apoptosis via the activation of caspases in neuroectodermal cells (Fulda *et al*, 1997). However, the mechanism by which apoptosis is induced in B16F10 cells by BA remains to be determined. Cell arrest, damage to DNA or stress to the cytoplasm or cell membrane may be the cause of apoptosis (Aller *et al*, 1992; Gohen *et al*, 1992; Ko *et al*, 1997). We believe that results of analysis of cell cycle distributions or apoptotic pathways to determine mechanisms of cytotoxicity of other chemotherapeutic agents used in the treatment for malignant melanoma will open the gate for the establishment of effective combinational therapeutic regimens against the still untreatable malignant disease using BA.

We performed animal experiments to determine whether BA and VCR show a suppressive effect on the growth or metastatic state of B16F10 melanoma cells *in vivo*. Antimetastatic activity was investigated by counting lung metastatic nodules as reported previously (Jani *et al*, 1993; Kang *et al*, 2000; Feleszko *et al*, 2002), because B16F10 melanoma cells preferentially induce lung metastasis (Jani *et al*, 1993; Gude *et al*, 2001; Malafa *et al*, 2002). Although treatment with VCR alone resulted in a significant suppression of metastasis of melanoma cells to the lung, the additional use of BA resulted in greater suppression than that in the case of treatment with VCR alone. Treatment with a combination of VCR and BA resulted in a significant reduction in the numbers of lung nodules of more than 1 mm in diameter in mice compared with that in the case of treatment with VCR or BA alone. These results suggest that BA potentiates the antitumour activity of VCR also *in vivo*. For the establishment of metastasis, tumour cells must leave the original tumour mass, invade the surrounding tissue and then enter blood vessels. The next steps are tumour cell attachment to endothelial cells in the blood vessel, invasion and growth in the target organ (Liotta, 1986; Nicolson, 1988). We could not determine which metastatic steps were mainly inhibited by the combinational treatment with BA and VCR; however, the significant reduction in the number of lung nodules of more than 1 mm in diameter in the combinational treatment group indicates their inhibitory effect on tumour growth after attachment to target metastatic sites. Furthermore, we observed no significant difference between weight losses in the groups treated with VCR, BA and both, indicating that the combination of BA and VCR might be an effective regime with side effects of relatively small magnitude for the treatment of malignant melanoma.

In summary, treatment with a combination of VCR and BA resulted in synergistic suppression of cell growth *in vitro* and in the prevention of B16F10 melanoma experimental metastasis by direct suppression of cell growth *in vivo*. It is expected that BA can augment antitumour activities of various drugs showing different types of cytotoxic mechanisms.
